# Development of keratin nanoparticles for controlled gastric mucoadhesion and drug release

**DOI:** 10.1186/s12951-018-0353-2

**Published:** 2018-03-19

**Authors:** Zhongjun Cheng, Xiaoliang Chen, Dongliang Zhai, Feiyan Gao, Tingwang Guo, Wenfeng Li, Shilei Hao, Jingou Ji, Bochu Wang

**Affiliations:** 1 Key Laboratory of Biorheological Science and Technology, Ministry of Education, College of Bioengineering, Chongqing University, Chongqing, 400030 China; 2 College of Chemistry and Chemical Engineering, Chongqing University, Chongqing, 400030 China; 3 Collaborative Innovation Center for Brain Science, Chongqing University, Chongqing, 400030 China; 4 Department of Nuclear Medicine, Chongqing Cancer Institution, Chongqing, 400030 China

**Keywords:** Kerateine, Keratose, Mucoadhesion, Controlled drug release, Bioavailability

## Abstract

**Background:**

Nanotechnology-based drug delivery systems have been widely used for oral and systemic dosage forms delivery depending on the mucoadhesive interaction, and keratin has been applied for biomedical applications and drug delivery. However, few reports have focused on the keratin-based mucoadhesive drug delivery system and their mechanisms of mucoadhesion. Thus, the mucoadhesion controlled kerateine (reduced keratin, KTN)/keratose (oxidized keratin, KOS) composite nanoparticles were prepared via adjusting the proportion of KTN and KOS to achieve controlled gastric mucoadhesion and drug release based on their different mucoadhesive abilities and pH-sensitive properties. Furthermore, the mechanisms of mucoadhesion for KTN and KOS were also investigated in the present study.

**Results:**

The composite keratin nanoparticles (KNPs) with different mass ratio of KTN to KOS, including 100/0 (KNP-1), 75/25 (KNP-2), 50/50 (KNP-3), and 25/75 (KNP-4), displayed different drug release rates and gastric mucoadhesion capacities, and then altered the drug pharmacokinetic performances. The stronger mucoadhesive ability of nanoparticle could supply longer gastric retention time, indicating that KTN displayed a stronger mucoadhesion than that of KOS. Furthermore, the mechanisms of mucoadhesion for KTN and KOS at different pH conditions were also investigated. The binding between KTN and porcine gastric mucin (PGM) is dominated by electrostatic attractions and hydrogen bondings at pH 4.5, and disulfide bonds also plays a key role in the interaction at pH 7.4. While, the main mechanisms of KOS and PGM interactions are hydrogen bondings and hydrophobic interactions in pH 7.4 condition and were hydrogen bondings at pH 4.5.

**Conclusions:**

The resulting knowledge offer an efficient strategy to control the gastric mucoadhesion and drug release of nano drug delivery systems, and the elaboration of mucoadhesive mechanism of keratins will enable the rational design of nanocarriers for specific mucoadhesive drug delivery.

**Electronic supplementary material:**

The online version of this article (10.1186/s12951-018-0353-2) contains supplementary material, which is available to authorized users.

## Background

Mucoadhesion may be defined as the state in which two materials, between which at least one is biological in nature, adhere to each other for extended retention time by the establishment of interfacial bonding [1, 2]. Nanotechnology-based drug delivery systems have been widely used for oral polymeric dosage forms and systemic drug delivery depending on the mucoadhesive interaction [3–5], which have the potential to increase residence time of drug delivery at particular body sites and tissues, reduce administration frequency and improve drug bioavailability [6]. In particular, mucoadhesive drug delivery systems have been used to prolong the gastric residence time of formulations, especially for drug substances whose targets are in the stomach with a narrow absorption window, or which are unstable or poorly soluble in the intestinal environment [7].

Many polymers, including chitosan, alginate, carboxypolymethylene, crosslinked polyacrylic acids, carboxymethyl cellulose and hydrophobin, are widely used for various drug delivery that rely on their mucoadhesive properties [1, 3]. The poly (isobutylcyanoacrylate)/thiolated chitosan nanoparticles were developed to enhance the intestinal permeability of docetaxel due to their mucoadhesion [8]. Hydrophobin-coated porous silicon nanoparticle was prepared and retained by gastric mucoadhesion in the stomach up to 3 h after oral administration [7].

Keratins, categorized as intermediate filaments and the cytoskeletal components of desmosome cellular junctions, have attracted great interest in biomedical applications [9]. Human hair keratins have been used for nerve regeneration, wound healing, hemostasis, bone regeneration and cell culture due to their excellent biocompatibility, biodegradability, non-immunogenic nature, cellular attachment, proliferation and viability features [10–12]. However, to our knowledge, no reports have been published about the mucoadhesive properties and mechanisms of keratins.

Keratins are processed as oxidized and reduced forms in term of keratose (KOS) and kerateine (KTN) according to the different extraction methods [13]. The cysteine sulfur atoms of KTN and KOS is in the form of thiol groups and sulfonic acid groups, respectively [14], which results in different properties of KOS and KTN with respect to mucoadhesion, hydrophilicity, solubility, surface charge and degradation rate [11, 14, 15]. Therefore, we assume that the KTN and KOS would display different mucoadhesive properties and possess different mechanisms of mucoadhesion, which can be used for the fabrication of mucoadhesion controlled keratin-based drug delivery systems. Thus, the mucoadhesion controlled keratin nanoparticles (KNPs) with different ratios of KOS to KTN were prepared to achieve controlled gastric mucoadhesion and drug release. Amoxicillin (AMO) was selected as the model drug. The influences of KNPs with different proportion of KTN and KOS on in vitro drug release, in vivo gastric retention time and bioavailability were investigated. Furthermore, the mucoadhesive mechanism of keratins at different pH conditions were also studied via the atomic force microscopy (AFM), surface contact stage, zeta potential and particle size, isothermal titration calorimetry (ITC), and turbidimetric titration measurements.

## Experimental section

### Materials

Human hair was purchased from local barber shops in Chongqing, China. Porcine gastric mucin (PGM) was purchased from Sigma-Aldrich. (UK). *N*-ethylmaleimide (NEM) was purchased from Macklin Biochemical Co. Ltd. (Shanghai, China). Urea, Tween 20, Thioglycolic acid (TGA) and glacial acetic acid were purchased from Kelong Chemical Reagent Co. Ltd. (Chengdu, China). Amoxicillin sodium (AMO) was supplied by the Tuochukangyuan Pharm & Chem Co., Ltd. (Hubei, China). All other materials and reagents used in this study were analytical grade.

### Mucoadhesive KNPs preparation

KNPs were prepared via the ultrasonic dispersion method based on our previous method [16], and the schematic diagram of the ultrasonic system for the synthesis of KNPs is shown in Additional file 1: Figure S1. Firstly, KTN and KOS were dissolved into ultrapure water with different weight ratios of KTN to KOS, including 100/0 (KNP-1), 75/25 (KNP-2), 50/50 (KNP-3), and 25/75 (KNP-4). 0.15% keratin solutions (w/v) were injected into the diluted HCl solution (pH 3.0) under sonication via a needle and supplied by a syringe pump with the speed of 0.25 mL/min. The ultrasonication was performed by an ultrasonic cell disruption system (JY92-II, Scientz, China), and the power of ultrasonic cell disrupter was selected as 400 W. The resultant nanoparticles were lyophilized by a freeze dryer (230, Modulyod, USA) overnight and stored. In addition, the AMO-loaded KNPs were also prepared as above method, and AMO was added into the keratin solution at a concentration of 0.06% (w/v).

### Mucoadhesive KNPs characterization

#### Morphology

The surface morphology of the AMO-loaded KNP-3 were observed by scanning electron microscope (SEM). A piece of aluminum foil loading with KNP-3 was coated with gold metal under vacuum and then examined by SEM (Nova 400, FEI, USA).

#### Particle size and zeta potential measurements

The particle size distribution, polydispersity index (PDI) and zeta potentials of the KNPs were measured using a Zetasizer (Nano ZS90, Malvern, UK). 0.1 N HCl solution (pH 1.2) was prepared as simulated gastric fluid (SGF), which was used to dilute the samples with appropriate concentrations prior to detection. In addition, the stability of KNPs was also evaluated by monitoring the particle size within 7 days.

#### Drug loading capacity measurement

The loading capacity (LC) and entrapment efficiency (EE) of the AMO-loaded KNPs were determined by following method. KNPs were incubated with the sodium hydroxide solution (pH 10.0) overnight, and the amount of AMO in the solution was detected using a UV spectrophotometer at 230 nm (Lambda 900, PerkinElmer, USA). The loading capacity and entrapment efficiency were calculated by following Eqs. (1) and (2):

1 $${\text{LC}} = \frac{{{\text{Weight}}\;{\text{of}}\;{\text{AMO in KNPs}}}}{\text{Weight of KNPs}} \times 100 {\text{\% }}$$

2 $${\text{EE}} = \frac{\text{Loading capacity of KNPs}}{\text{Loading capacity of KNPs in theory}} \times 100 {\text{\% }}$$

### FT-IR studies

The chemical structure and complex formation of AMO, KTN, KOS, and AMO-loaded KNPs were analyzed by FT-IR (5DX/550, Nicolet, USA). The samples were prepared by grinding the dry specimens with KBr and pressing them to form disks.

### In vitro drug release studies

The in vitro release study of AMO from KNPs was also carried out. The KNPs and 3 mL SGF (pH 1.2) were put into a dialysis tube (MWCO: 12,000), and the dialysis tube was placed in 30 mL SGF at 37 °C and maintained under shaking at 100 rpm (sink condition). At specific time intervals, sample (1 mL) was taken and replaced with fresh SGF. The concentration of AMO was determined using a UV spectrophotometer (Lambda 900, PerkinElmer, USA).

### Gastric retention tests

Iodine-131 (^131^I) was selected to radiolabel the AMO-loaded KNPs because of its long half-life (8.02 days). Radiolabeling of the KNPs was carried out by direct labelling method (physical adsorption). AMO-loaded KNPs were put into a tube, and an aliquot of ^131^I solution equivalent to radioactivity of 5 mci was added into the tube. The labelled KNPs were recovered by filtration through a filter paper and dried at room temperature overnight. All operations were performed in fume hood by a nuclear medicine specialist. The stability of ^131^I-labelled KNPs was also tested following the previous study [17].

Male SD rats weighing approximately 250–300 g were used in the study. No rats were taking any regular medication or had a history of gastro-intestinal disorders. After fasting for 24 h prior to experiment, the ^131^I-labelled KNPs (30 μci) were administered through an oral feeding needle. Scintigrams of test preparation were recorded by a single-photon emission computed tomography (SPECT) apparatus with variable angle dual-detector units (Symbia T2, Siemens, USA). The biodistribution of ^131^I-labelled KNPs in vivo was scanned at the end of gamma scintigraphy studies using a hybrid scanner comprising a two-row spiral CT and a SPECT camera (Symbia T2, Siemens, USA). After the SPECT data was acquired immediately, the raw data were reconstructed into transverse slices using a workstation (Siemens Medical Solutions). A region of interest (ROI, a circle with 100 mm diameter) was drawn to determinate the radioactivity in the stomach at different time points. ROI Quantification was performed with the syngo^®^ MI Acquisition Workplace (Siemens Medical Solutions). The radioactivity in the ROI at 0 h was taken as the control and designated as 100%.

### Pharmacokinetic tests

Male SD rats weighing 250–300 g were fasted for 12 h before drug administration. Each formulation was dispersed in deionized water prior to dosing and administered by oral gavage at a dose of 37.575 mg/kg of AMO. Blood samples were withdrawn from retro orbital choroid plexus under mild anesthesia at 0, 0.25, 0.5, 1, 1.5, 2, 4, 6, 8, 10, and 12 h after dosing and placed into heparin pretreated tubes. The blood samples were centrifuged at 5000*g* for 10 min and plasma was stored at − 20 °C until further analysis. Plasma concentration of AMO, was determined according to a validated HPLC method [18]. Samples were analyzed using the Agilent HPLC system equipped with an Ultimate XB-C18 column (250 × 4.6 mm, 5 μm, 120 Å) maintained at 37 °C. The mobile phase was a mixture of 10 mM phosphate buffer (pH 6.0) and acetonitrile (80:20, v/v) at the flow rate of 1.0 mL/min. The UV detector was set to 228 nm.

The pharmacokinetic parameters, including the area under the plasma concentration–time curve from 0 to 12 h (AUC_0–12 h_), time to reach maximum plasma concentration (T_max_), and the peak plasma concentration of drug (C_max_) after administration of KNPs in SD rats were determined using a one-compartmental analysis by a freely available add-in program for Microsoft Excel, PKSolver.

In addition, the in vivo toxicity of prepared keratins nanoparticles (KNP-3, 380 mg/kg) were also studied by intragastric administration for 7 days. The animals were anaesthetized, and the main tissue organs of the rats were then fixed in 4% paraformaldehyde for histopathologic examination.

### Interaction mechanisms of keratins with PGM

The ability of keratins (KTN and KOS) to interact with PGM were also investigated using the atomic force microscopy, sizes and zeta potential, surface wettability, ITC and turbidimetric analyses in the present study.

The atomic force microscopy observation were performed as follow: KTN and KOS were extracted from the human hair as described by our previous studies [16, 19]. KTN, KOS, PGM, the mixture of KTN and PGM, and the mixture of KOS and PGM (1:1, w/w) were diluted with phosphate buffer (pH 7.4 and 4.5) and SGF (pH 1.2) to 2–4 mg/mL. An aliquot (2 μL) of the diluted sample solutions was spread on freshly cleaved mica surfaces and then dried at ambient temperature. Tapping mode was carried out with a probe constructed from silicon using a multimode NanoScope IIIa AFM (Digital Instruments, USA), and the quoted spring constant and resonant frequency was 20–80 N/m and 307 ± 375 kHz, respectively. The AFM Gwyddion software was used to analyze the recorded scans.

The sizes and zeta potential measurements were also used to assess the interaction between keratins and PGM and carried out as follow: KTN, KOS and PGM were separately dispersed in PBS solutions (pH 7.4, and 4.5) at a concentration of 1% w/v, and then the concentrations of KTN, KOS and PGM were diluted with corresponding media to make the final concentration of 0.5–0.01% w/v. The KTN–PGM mixture and KOS–PGM mixture were prepared in different proportions of PGM, and the size and zeta potential measurements for all formulations were conducted using a zetasizer (Nano ZS90, Malvern, UK).

The surface wettability of KTN and KOS was measured by contact angle measurement (SDC-200, Shengding Precision Instrument Co., Ltd, China). The contact angle measurements were carried out using photology system equipped with microscope. A drop of SGF (pH 1.2, 15 μL) was dropped onto the surface of KTN or KOS-coated glass slides, and the measured results were calculated and recorded by software. The recorded contact angles were the averages of six measurements made on different areas of the surface.

An ITC-200 titration microcalorimeter (MicroCal, Inc., Northampton, MA) was used at 37 °C. PGM solutions (1%, w/v) were ultra-centrifuged at 50,000 rpm for 20 min at 4 °C to discard aggregate and ensure a stable heat flow. KNT and KOS solutions were prepared at 1% (w/v). The pH of all solutions was adjusted to 4.5 and 7.4. PGM solution (40 μM) was placed in the 200 μL sample cell, and KTN and KOS solutions were loaded into the injection syringe, respectively. Both solutions were degassed before each titration. The duration of each injection was 5 s, and the time interval between injections was 120 s. The solution in the cell was stirred at 750 rpm by the syringe to ensure thorough mixing. The interaction enthalpy, entropy, binding equilibrium constant, and stoichiometry number were calculated from the titration curve using MicroCal Origin fitting software version 7.0.

Turbidimetric measurements were performed spectrophotometrically. PGM solutions (0.4%, w/v) were prepared as the ITC analysis described, and titrated with 0.4% (w/v) of KTN and KOS at pH 4.5 and 7.4. The absorbances of mixture samples were recorded using a UV–Vis spectrophotometer at 520 nm. Samples were measured as controls and the turbidity (τ) calculated by the Eq. τ = − 1/L ln (I/I0), where L is the path length (1 cm), (I/I0) = 10 − A, and A is absorbance at λ = 520 nm. In addition, the effects of urea, Tween 20, and *N*-ethylmaleimide (NEM) on the chemical interactions between keratins (KTN and KOS) and PGM were also investigated via turbidimetric measurements. Turbidity of keratins and PGM mixtures (1:1 w/w, based on solution amount) at pH 4.5 and 7.4 was measured before and 1 h after adding one of the three following chemical blockers: urea (6 M) for hydrogen bonding, Twee n 20 (1%, w/v) for hydrophobic interactions and NEM (6 mM) for disulfide bonds.

### Statistical analysis

All measurements were performed at least in triplicate and data were presented as mean ± standard deviation (SD). Three batches of nanoparticles were prepared for each formulation. For selected evaluation tests, the means of all tested formulations were compared with each other by means of a one-way ANOVA with Tukey’s post hoc comparisons. The statistical significance level (*P*) was set at < 0.05.

## Results and discussion

### KNPs preparation and characterization

The earliest record about keratins for biomedical application was from a Chinese medical book called Ming Yi Bie Lu in the 5th century, which referred that burnt human hair could astringe leakage of blood and stop bleeding [16]. In the recent decades, keratin-based biomaterials have been prepared as the films, hydrogels, dressing and scaffolds, which were used in a variety of biomedical applications, including nerve regeneration, wound healing, bone regeneration and cell culture due to their intrinsic biocompatibility, biodegradability, and natural abundance [10, 13, 14, 16]. However, few studies focused on the development of keratin-based mucoadhesive drug delivery, and the mechanism of mucoadhesion of keratins is also not yet clear. Therefore, we have fabricated the mucoadhesion controlled keratin nanoparticles an d investigated their mucoadhesive mechanisms.

Based on the different hydrophilicity, solubility, surface charge and degradation rate between KTN and KOS, which may possess different mucoadhesive abilities. The mucoadhesion controlled KTN/KOS composite nanoparticles can be fabricated by adjusting the mass ratio of KTN to KOS, and the drug release rate of nanoparticle can also be controlled due to their different pH-sensitive properties. The composite keratin nanoparticles with different mass ratio of KTN to KOS, including 100/0 (KNP-1), 75/25 (KNP-2), 50/50 (KNP-3), and 25/75 (KNP-4), were prepared via the ultrasonic dispersion method, and the schematic diagram of the ultrasonic system for the synthesis of KNPs is shown in Additional file 1: Figure S1 [20]. The KTN aggregates can be formed in the acid medium (pH < 3.5), and ultrasonic was used to disperse the particle. In addition, the sustained drug release effect of KOS is weak in the stomach due to its solubility, so we did not prepare and evaluate the pure KOS nanoparticles in this study.

Table 1 shows the effect of different ratios of KTN to KOS on the characterizations of AMO-loaded KNPs. The loading capacity (LC) of AMO-loaded KNPs decreased from 15.73 to 8.88% with decreasing the ratio of KTN to KOS. The increase of KOS proportion led to a decrease in entrapment efficiency (EE), which could be explained by the fact that the formation of KNPs was attributed to the KTN agglomeration in acidic solution, but the addition of KOS into KNPs would result in the drug leakage. The particles size of KNPs decreased linearly from 395.3 to 345.5 nm with decreasing the ratio of KTN to KOS, which was also due to the agglomeration of KTN. Furthermore, the time-dependent changes in the particle size of KNPs were investigated (Additional file 1: Figure S2), the no significant increase in average hydrodynamic size of KNPs were observed with increasing time. The results indicated that prepared KNPs restored its colloidal stability within 7 days. Thus, the KTN/KOS mixed particles could be more easily dispersed in the acid solution using ultrasonic compared to the pure KTN particles. Furthermore, KTN and KOS nanoparticles displayed the positive and negative surface charge in the simulated gastric fluid (SGF) respectively, and a gradual decrease in zeta potential of KNPs was noted with the increase of KOS proportion.

**Table 1:** Effect of different ratios of KTN to KOS on the characteristics of AMO-loaded KNPs (mean ± SD; *n* = 3)

Formulation	AMO (%)		Size		Zeta potential (mV)
	LC	EE	nm	PDI	
KNP-1 (100/0)	15.73 ± 0.44	62.65 ± 1.99	395.3 ± 21.5	0.240 ± 0.020	14.25 ± 0.23
KNP-2 (75/25)	11.01 ± 1.03	59.49 ± 0.83	370.0 ± 19.6	0.300 ± 0.077	9.74 ± 0.15
KNP-3 (50/50)	9.89 ± 0.16	53.01 ± 2.11	354.6 ± 20.9	0.251 ± 0.042	7.65 ± 0.27
KNP-4 (25/75)	8.88 ± 1.20	49.96 ± 0.98	345.5 ± 15.3	0.242 ± 0.050	− 3.23 ± 0.41

**Fig. 1:**
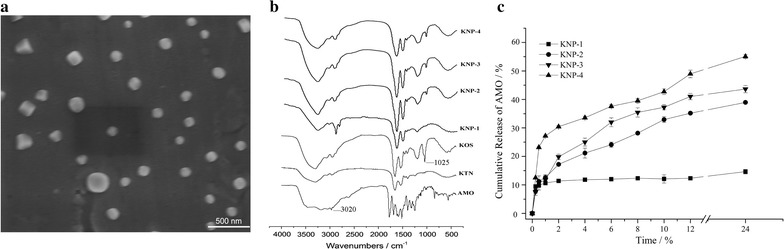
**a** SEM image of KNP-3. **b** FT-IR spectra of KTN, KOS, AMO and KNPs. **c** Release profiles of AMO from KNPs in SGF

The morphological characteristics of the KNP-3 were observed using scanning electron microscope (SEM) and the results are shown in Fig. 1a. The average size ranged from 200 to 400 nm, which was consistent with the results of particle size detection.

The FT-IR spectra of KTN, KOS, AMO and KNPs are shown in Fig. 1b. The spectra of keratins (KTN and KOS) exhibited several characteristic absorbance bands of proteins that assigned to the peptide bonds (–CONH–), known as amide A (2800–4000 cm^−1^), amide I (1600–1700 cm^−1^), amide II (1480–1580 cm^−1^) and amide III (1220–1330 cm^−1^) peaks [10]. The symmetric stretching vibration of cysteine-*S*-sulfonate residue at 1025 cm^−1^ was found in the FT-IR spectra of KOS as well as in the FT-IR spectra of KNP-2, KNP-3 and KNP-4, suggesting that KOS was introduced into the KTN nanoparticles. Meanwhile, the relative intensity of absorbance band at 3020 cm^−1^ corresponding to benzene ring CH stretch for KNPs increased compared to KTN and KOS [21], which might result from the entrapment of AMO into KNPs. However, no new band was found in the IR spectra of KNPs, suggesting that no changes in chemical conformation of keratins and drug within the KNPs [22].

### In vitro drug release studies

The release profiles of AMO from KNPs were investigated in SGF (Fig. 1c), and the release rate of AMO could be adjusted by changing the ratios of KTN to KOS in KNPs. KNP-1 (pure KTN) showed slow AMO release during 24 h, which resulted from the low solubility of KTN in acidic solution (pH < 3.5). In addition, the release rate of AMO increased with an increase in KOS proportion within the KNPs. Enhanced drug release is a result of higher particle hydration (higher diffusion coefficient for drug) or faster degradation/dissolution of the particle when more of the hydrophilic KOS is added. Thus KNP-4 (75% of KOS) displayed the fastest drug release among the four KNPs, and approximately 55% AMO were released in the SGF for 24 h. Therefore, the drug release rate can be controlled by changing the ratios of KTN to KOS in the present study.

### Gastric retention tests

**Fig. 2:**
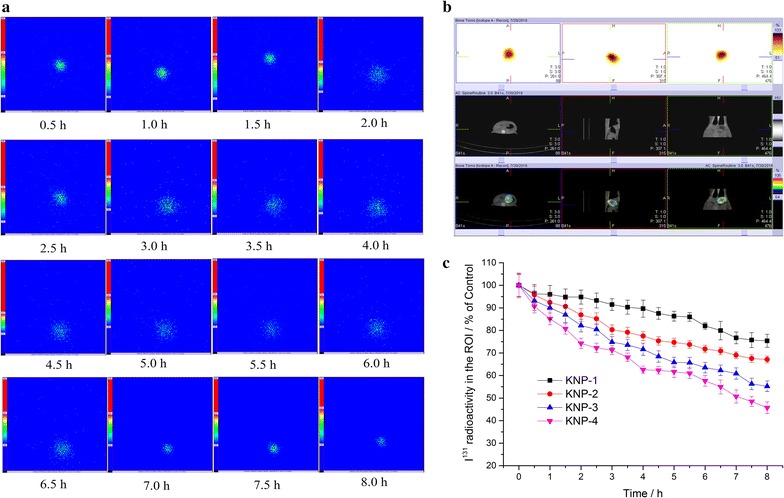
**a** Gammascintigraphic images of ^131^I labeled KNP-3 in rat. **b** Biodistribution of ^131^I-labeled KNP-3 after oral administration for 8 h in rat. Top row localization CT; middle row SPECT images; bottom row fused co-registered images. **c** Radioactive counts per 2 min of ^131^I-labeled KNPs in the ROI. The values are mean ± SD (*n *= 6)

The in vivo mucoadhesive property of prepared KNPs can be evaluated by detecting their gastric retention time in rats. Iodine-131 (^131^I) was used to radiolabel the AMO-loaded KNPs via physical adsorption as described in our previous studies [22, 23]. The stabilities of ^131^I-labeled KNPs in standard buffer solutions of pH 1.2, 4.5 and 7.4 were evaluated. The activity released from ^131^I-labeled KNPs was less than 5.0% in pH 1.2, 6.0% in pH 4.5, and 8.0% in pH 7.4 within 8 h, respectively. Figure 2a shows the gamma scintigraphic images of the fasted rats after the oral administration of ^131^I-labeled KNP-3 within 8 h. An intense hotspot could be observed in the stomach, indicating that KNP-3 remained in the stomach. However, the radioactivity decreased over time due to the peristalsis of stomach. The in vivo biodistribution study is a useful supplement for the gamma scintigraphy study and could provide anatomic localization of hotspots in hybrid imaging using SPECT/CT techniques. Figure 2b shows the planar SPECT (top row), CT (middle row) and SPECT/CT fused images (bottom row) of the rat after the oral administration of ^131^I-labeled KNP-3 for 8 h. In order to identify the hotspot location, the images were reconstructed into transverse, sagittal, and coronal slices, and the results indicated that the hotspot observed in the gamma scintigraphy was derived from the ^131^I-labeled KNP-3 in the stomach. Furthermore, ROI (a circle with 100 mm diameter) was also drawn manually around the stomach to measure the radioactive counts in the stomach [23]. Figure 2c displays the radioactive counts per 2 min of ^131^I-labeled KNPs in the ROI within 8 h. The gastric retentive ability of KNPs decreased with the increase of KOS proportion within the KNPs. However, more than half of the KNPs were maintained in the stomach of rats up to 8 h, which demonstrated that the KNPs had good gastric retention and the gastric retentive capacity could be adjusted by changing the ratios of KNT to KOS.

### Pharmacokinetic tests

**Fig. 3:**
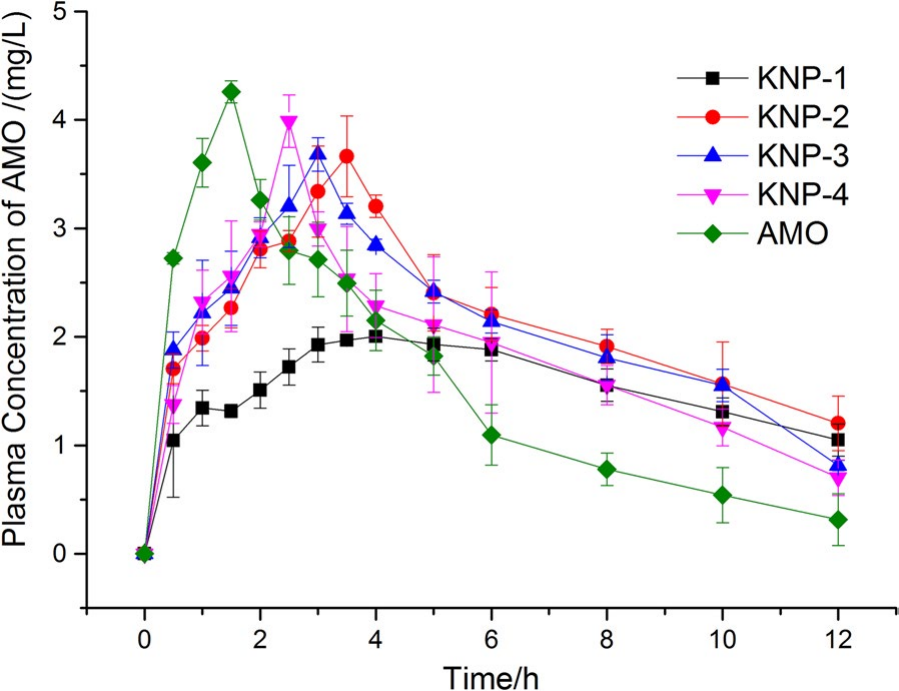
The plasma concentration–time profiles of KNPs and pure AMO (*n *= 6)

The oral drug bioavailability usually can be controlled by adjusting the in vivo drug release and gastric retention time. Thus, the in vivo PK performances of KNPs and pure AMO were also assessed in this study. The plasma concentration–time profiles are shown in Fig. 3 and the PK parameters in Table 2. Pure AMO was absorbed quickly after oral administration, and a maximum plasma concentration (C_max_) reached approximately 3.75 ± 0.11 μg/mL at 1.26 h. Compared to pure drug, the C_max_ of AMO from KNPs became lower and the time to maximum plasma concentration (T_max_) increased due to the sustained release of AMO and prolonged gastric retention time. Among the four nanoparticles, KNP-1 had the highest T_max_ of 3.75 ± 0.12 h and the lowest C_max_ of 1.95 ± 0.01 μg/mL. This was probably due to the slowest release rate of AMO, and less than 15% of drug released within 12 h from KNP-1 in vitro. Thus, KNP-1 exhibited a significant lower area under the plasma drug concentration–time curves from time zero to 12 h (AUC_0–12 h_) than other KNPs (P < 0.05). Furthermore, pharmacokinetic parameters were altered depending upon the nanoparticles used. Compared to KNP-1, the AUC_0–12 h_ of AMO was increased 1.4-, 1.3- and 1.2-fold for KNP-2, KNP-3, and KNP-4, respectively. These results clearly indicated that the KNPs with different ratios of KTN to KOS could achieve a controlled drug release rate and gastric retention time, and adjust the oral bioavailability of AMO.

**Table 2:** Pharmacokinetic parameters for AMO-loaded KNPs in rats after oral administration (mean ± SD; *n* = 6)

Formulation	T_max_ (h)	AUC_0–12 h_ (µg h/mL)	C_max_ (µg/mL)
KNP-1	3.75 ± 0.12	18.28 ± 1.35	1.95 ± 0.01
KNP-2	3.02 ± 0.08	25.82 ± 1.09	3.12 ± 0.33
KNP-3	3.00 ± 0.10	24.05 ± 1.17	3.23 ± 0.29
KNP-4	2.33 ± 0.02	22.10 ± 0.28	3.02 ± 0.10
AMO	1.26 ± 0.04	18.90 ± 0.10	3.75 ± 0.11

Moreover, the in vivo toxicity of prepared KNP-3 (380 mg/kg) were also studied by intragastric administration (Additional file 1: Figure S3). H&E staining of major organs was carried out to investigate the systemic toxicity of KNPs, and no significant histopathological differences of the organs were observed. The results indicated that no apparent toxicity to the studied animals was found after oral administration of the KNPs for 7 days. In addition, the keratin nanoparticles, hydrogel and fibers have been prepared and used for the hemostasis and other biomedical applications, and the in vivo biological safety of keratins have been investigated in our previous study [16]. Subcutaneous implantations into rats were performed to assess the in vivo toxicity of keratin fibers. No obvious organ damages of rats in implanted groups were observed via H&E staining, and no significantly elevation of serum levels of inflammatory cytokines including interleukin-1 beta (IL-1β), IL-6 and tumor necrosis factor-alpha (TNF-α) were found in the experimental groups [19].

### Interaction mechanisms studies of keratins with PGM

**Fig. 4:**
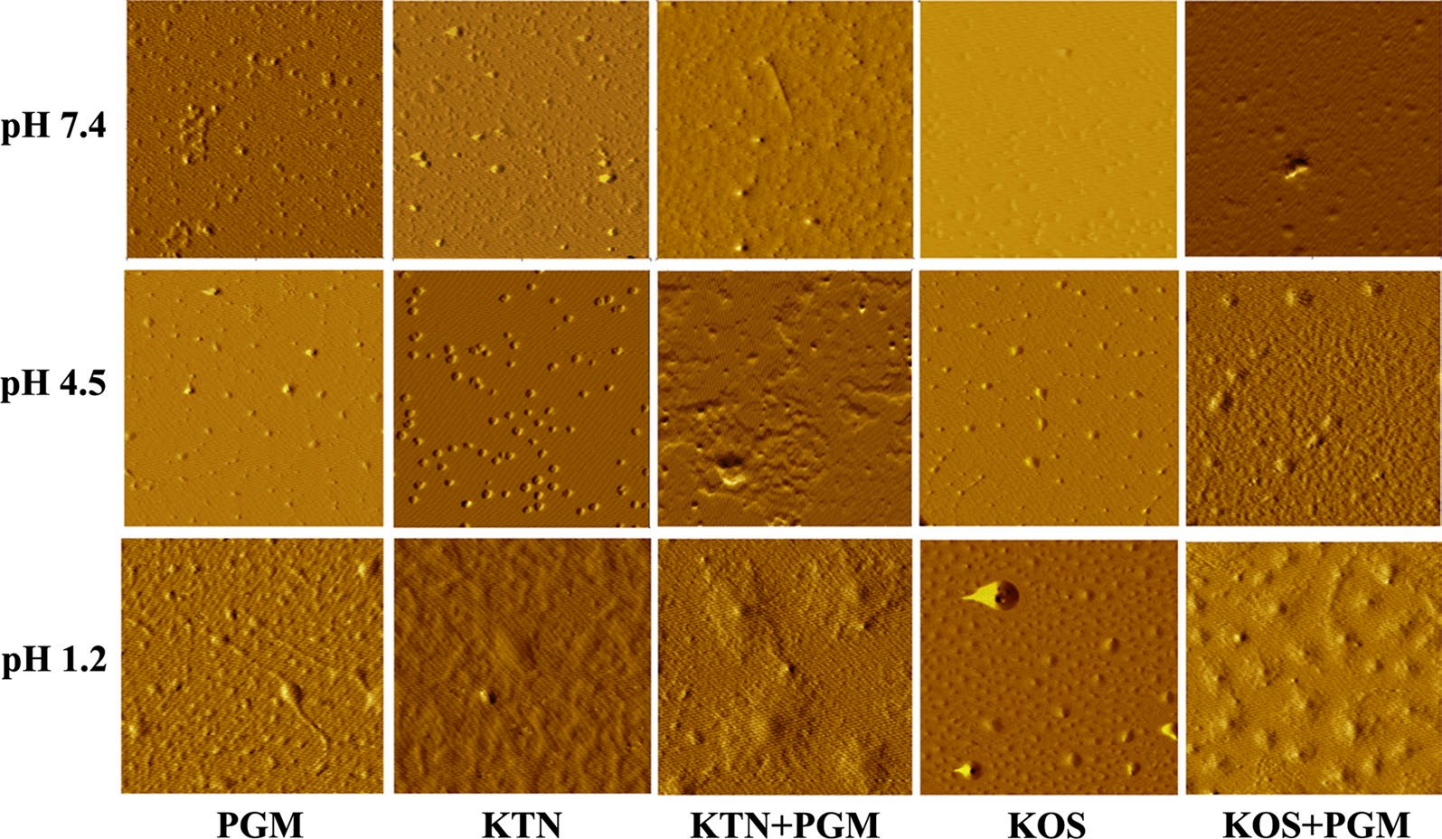
AFM observation of the interaction between PGM and keratins in different pH conditions. Image size is 2.0 × 2.0 μm^2^

Firstly, the visualization of keratins (KTN and KOS) adsorption on PGM molecules was directly revealed by the AFM and size and potential measurements. As shown in Fig. 4, PGM formed gel at low pH, but it could disperse at higher pH environment [24]. Thus, small PGM particles were observed at pH 7.4 and 4.5, and big PGM particle aggregates were formed at pH 1.2. A similar pH-sensitive property was also observed for KTN, and the formation of large KTN particle was due to its low solubility at pH 1.2. Whereas, KOS aggregates were absent in the AFM images at low pH (1.2 and 4.5), and a gradual increase in the particle size was noted with the decrease in pH value of solution. In addition, the particle size of keratins became bigger after mixing with PGM, which indicated that keratins had strong affinity to PGM in different pH buffer solutions, and the decrease of pH solution led to an increase in particle size of keratins–PGM complexes. Moreover, larger clump-like aggregates were observed after mixing of KTN and PGM compared to the KOS–PGM complex, indicating that KTN had a stronger affinity to PGM than KOS in different pH conditions.

**Fig. 5:**
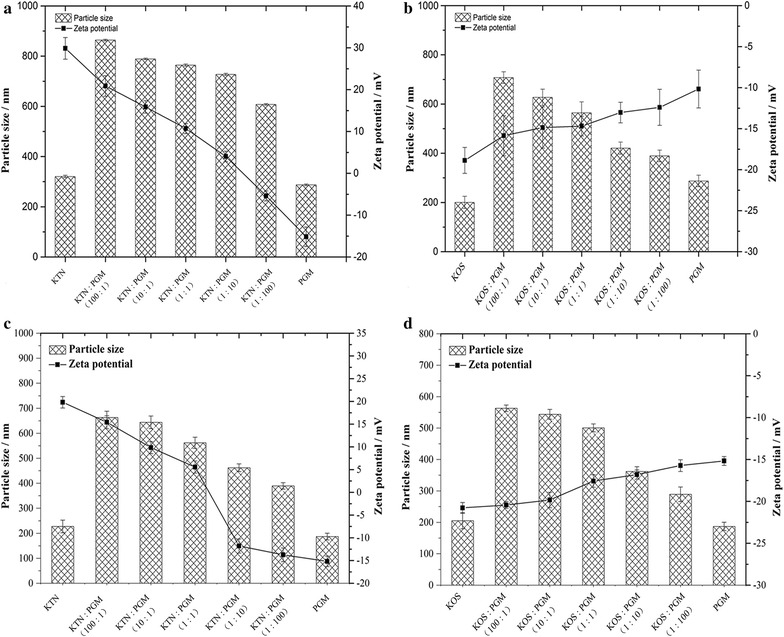
The zeta potential and mean particle size of KTN, KOS, PGM and the mixture in various ratios at pH 4.5 (**a**, **b**) and 7.4 (**c**, **d**)

In addition, the sizes and zeta potential measurements were also used to assess the interaction between keratins and PGM [25]. Figure 5a–d shows the zeta potential and mean particle size of KTN, KOS, PGM and the mixtures after mixing of keratins in various ratios at different pH (4.5 and 7.4). KTN displayed a positive surface charge, while the zeta potential of PGM and KOS were negative charge. As shown in Fig. 5a, the zeta potential of KTN–PGM complex in pH 4.5 condition decreased from + 20.87 to − 5.40 mV as the proportion of PGM increased, which indicated that KTN had a high affinity to PGM particles to cover their surfaces. It was expected that the surface property of the PGM particles might be changed by the adhesion of the polymer if the polymer had a mucoadhesive property. Furthermore, the zeta potential of PGM particle also changed after the addition of KOS into the PGM solution at pH 4.5 (Fig. 5b). The zeta potential of KOS–PGM complex was shifted to lower negative value in the case of the solution containing higher proportion of PGM. A prominent pH gradient was established in the gastric mucus from the lumen (pH 1–2) to the epithelial cell (pH 6–7) [26], so the zeta potential of KTN–PGM and KOS–PGM complex was also assessed at pH 7.4. As the proportion of PGM increased, a decrease in the zeta potential of KTN–PGM complex was observed from 15.43 to − 13.73 mV (Fig. 5c), while no marked change was observed for KOS–PGM complex (Fig. 5d). This was attributed to the similar values for KOS and PGM in pH 7.4 buffer.

Furthermore, a modified PGM particle method was also performed to measure the mucoadhesive interaction as described in this study (Fig. 5a–d) [27]. The mean particle size of PGM was approximately 298 and 198 nm in buffer solutions at pH 4.5 and pH 7.4, respectively, and that of KTN at was 320 nm at pH 4.5 and 227 nm at pH 7.4. After mixing KTN with PGM, the particle size of KTN–PGM with different proportions of KTN in the mixture complexes significantly increased compared to that of pure PGM due to the electrostatic interactions, suggesting that KTN had a strong affinity to PGM in pH 4.5 and 7.4 buffer solutions [25, 28]. The similar results were observed when KOS mixed with PGM, but electrostatic interactions would not take part in the interaction between KOS and PGM due to the same surface charges. In addition, the particle size of KTN–PGM complex was larger than that of KOS–PGM complex at the same ratios of keratins to PGM. The results revealed that KTN displayed a stronger affinity to PGM than KOS in the above solutions. Overall, the results of AFM observation and size and zeta potential measurement indicated that keratins, including KTN and KOS, had strong affinity to PGM in different pH buffer solutions, and KTN shows a stronger affinity to PGM compare to KOS. Furthermore, more subsequent studies have been done to illustrate the mechanisms of mucoadhesive of keratins.

**Fig. 6:**
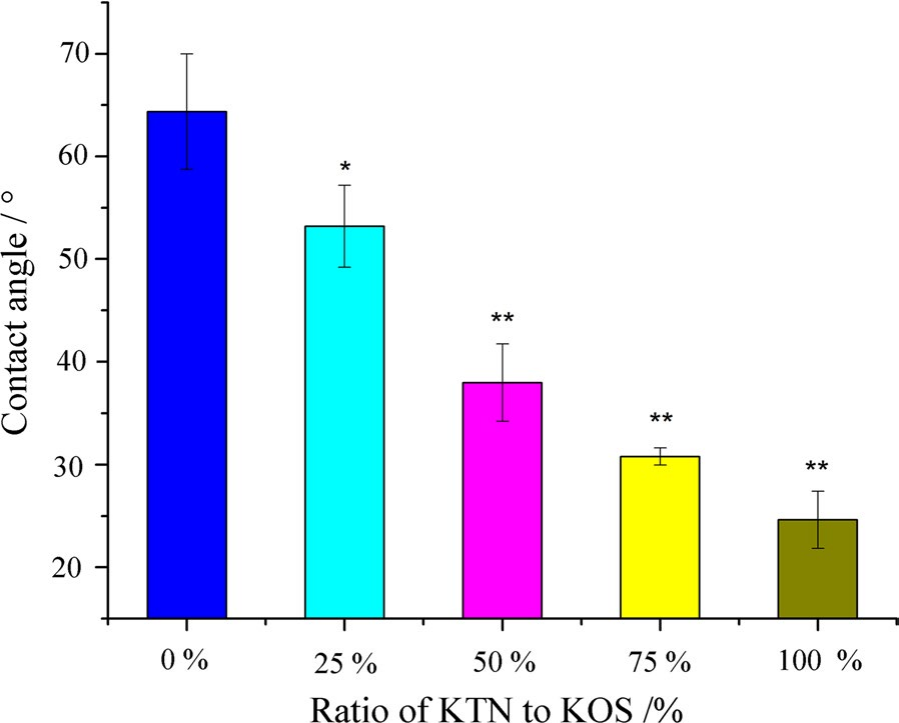
The contact angle of SGF to keratins with different mass ratios of KOS to KTN (n = 6). **P < 0.01, *P < 0.05 vs. pure KOS

The mucoadhesive process usually contains two stages, the contact stage followed by the consolidation stage [2]. Various physicochemical interactions, including hydrogen bondings, electrostatic attraction forces, disulfide bonds and hydrophobic effects, contribute to consolidate and strengthen the adhesive joint to prolonged adhesion [29, 30]. Therefore, the wettability of KTN and KOS were evaluated using contact angle goniometry. The wetting of SGF (pH 1.2) to keratins with different mass ratio of KOS to KTN was detected (Fig. 6), and the contact angle decreased from 64.35° to 25.05° with increasing mass ratio of KOS to KTN. This indicated that KTN and KOS are hydrophilic, and KOS had a higher hydrophilicity than KTN.

**Fig. 7:**
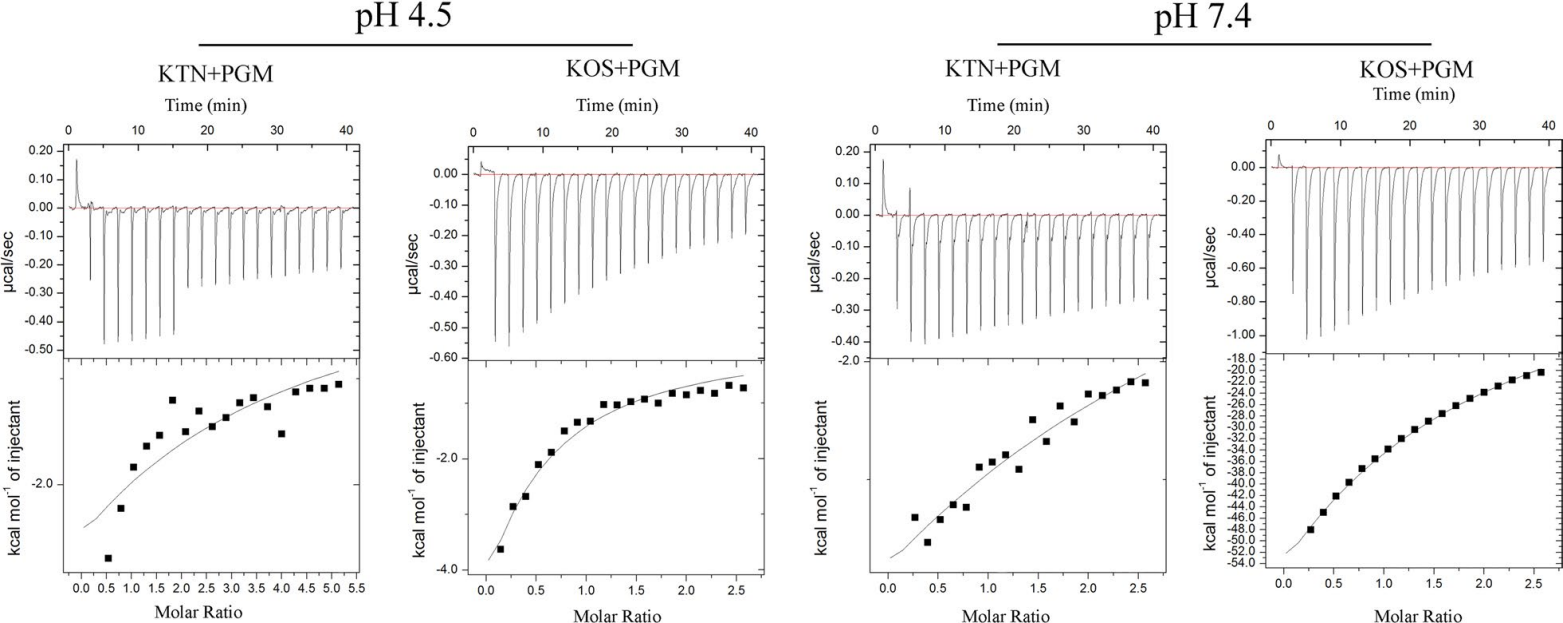
The ITC for binding of keratins (KTN and KOS) to PGM at pH 4.5 and 7.4

Furthermore, ITC is a versatile analytical method that has become a gold standard in characterization of molecular interactions, which allows quantitative determination of binding constants (Ka), reaction stoichiometry (*n*), enthalpy (∆H) and entropy (∆S) change, providing a complete thermodynamic profile of investigated interaction [31, 32]. 40 μM PGM dispersions were prepared at pH 4.5 and 7.4, and the heat flow was recorded when KTN or KOS was titrated into PGM solution (Fig. 7). Table 3 shows the thermodynamic parameters for binding of keratins (KTN and KOS) to PGM at pH 4.5 and 7.4. The association constant Ka (affinity, M^−1^) of KTN and KOS for PGM at pH 4.5 was 3.98 × 10^5^ and 1.55 × 10^4^, respectively, indicating a stronger affinity of KTN to PGM than KOS at pH 4.5. Whereas, the Ka values obtained with KTN–PGM and KOS–PGM complexes in pH 7.4 condition were 1.07 × 10^4^ and 4.14 × 10^4^, respectively. The affinity between KTN and PGM decreased as pH increased from 4.5 to 7.4, and KTN and KOS displayed a similar affinity to PGM at pH 7.4.

**Table 3:** Thermodynamic parameters for binding of keratins to MUC at different pH

Complexes	pH	*K*_*a*_ (M^−1^)	∆*H* (kcal/mol)	∆*G* (kcal/mol)	− *T*∆*S* (kcal/mol)	*n*
KTN + PGM	4.5	(3.98 ± 1.82) × 10^5^	− 2.41 ± 0.02	− 33.2 ± 0.13	30.8 ± 1.07	1.23 ± 0.02
KOS + PGM	4.5	(1.55 ± 0.71) × 10^4^	− 4.25 ± 0.05	− 24.8 ± 0.20	20.6 ± 0.39	1.15 ± 0.03
KTN + PGM	7.4	(1.07 ± 0.12) × 10^4^	− 3.47 ± 0.10	− 23.9 ± 0.16	20.4 ± 0.14	1.50 ± 0.01
KOS + PGM	7.4	(4.14 ± 1.15) × 10^3^	− 61.1 ± 1.53	− 21.5 ± 1.01	− 39.6 ± 1.13	1.27 ± 0.02

In addition, stoichiometry (*n*) indicating the number of binding site of keratins and PGM are also shown in Table 3, and the *n* values of KTN and KOS at pH 4.5 and 7.4 ranged from 1.15 to 1.50, indicating that the keratins possessed one binding site for PGM. Moreover, the changes in enthalpy and entropy associated with the reaction at pH 4.5 and pH 7.4 were also investigated. Binding of KTN to PGM at pH 4.5 and 7.4 were associated with favorable enthalpy (∆H = − 2.41 and − 3.47 kcal/mol). Combing with the negative values of binding energy (∆G) and unfavorable entropy (− T∆S = 30.8 and 20.4 kcal/mol), the results suggested the binding between KTN and PGM is dominated by hydrogen bonding [33]. The mechanism behind the interaction of KOS and PGM at pH 4.5 also is hydrogen bonding formation by distinguishing the values of ∆H, ∆G and − T∆S. While, the interaction between KOS and PGM at pH 7.4 was associated with favorable entropy (− T∆S = − 39.6 kcal/mol) and favorable enthalpy (∆H = 61.1 kcal/mol), which indicated that the bindings are dominated by hydrophobic interactions and hydrogen bonds [31]. Therefore, the results of ITC measurement demonstrated that hydrogen bonding is responsible for PGM and keratins interactions at pH 4.5 and 7.4, and KOS binding to PGM in pH 7.4 condition is initiated by hydrophobic interactions and hydrogen bonds.

**Fig. 8:**
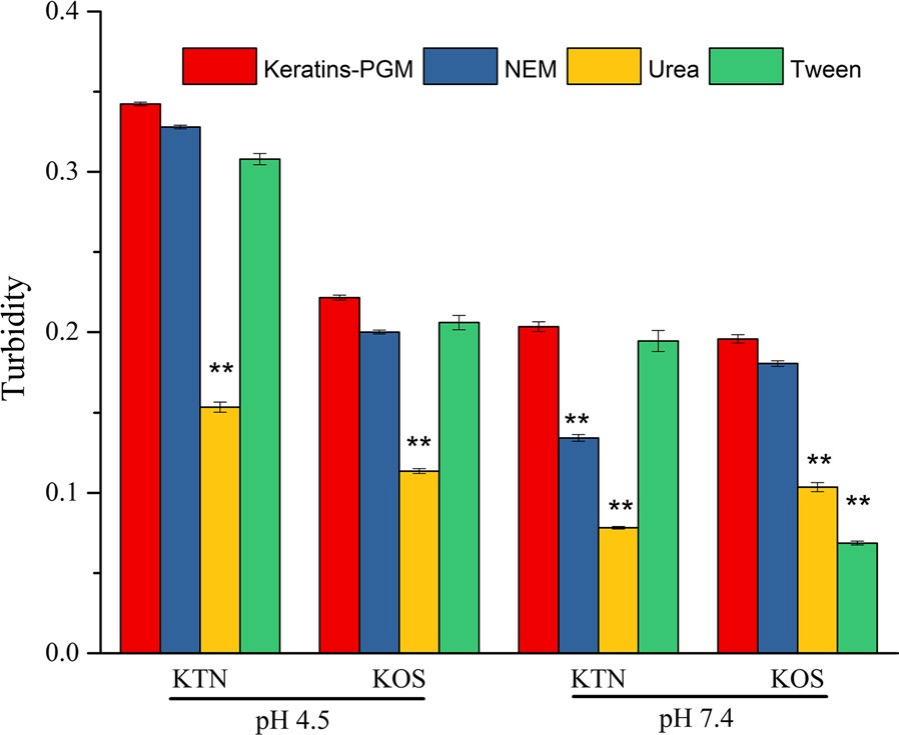
Turbidity of keratins–PGM solution after addition of different inhibitors of chemical interactions (*n* = 6). **P < 0.01, *P < 0.05 vs. control group

Moreover, the formation of a protein–protein rich phase gives rise to a sharp increase in turbidity of the solution, and a reduction of initial turbidity of protein–PGM mixture solution can be observed by addition of inhibitors, which was used to investigate the reactive mechanisms between protein and PGM [32]. Therefore, urea, Tween 20 and *N*-ethylmaleimide (NEM), acting as strong competitor for hydrogen bonding, hydrophobic interactions and disulfide bonds [34], were added into the KTN–PGM and KOS–PGM mixture solutions, respectively (Fig. 8). Reduction of turbidity of keratins–PGM solution was found when urea was added into the different keratin–PGM solutions, suggesting that hydrogen bonding contributes to the interactions between keratins (KTN and KOS) and PGM in pH 4.5 and 7.4 conditions. Furthermore, a highly significant reduction of turbidity of KOS–PGM solution at pH 7.4 was found in the Tween 20 solution (P < 0.01), confirming the presence of strong hydrophobic interactions. This was in agreement with the ITC measurements. Besides, keratins might establish disulfide bridges with cysteine residues of PGM, and the addition of NEM had significant influence on the turbidity of KTN–PGM solution at pH 7.4, indicating that disulfide bond plays a key role in the interaction between KTN and PGM in pH 7.4 condition.

Overall, the above studies related to the interaction between keratins and PGM indicated that hydrogen bonding plays a main role in the interaction between keratins (KTN and KOS) and PGM, and the electrostatic interactions and disulfide bondings take part in the KTN and PGM interaction at pH 4.5 and pH 7.4, respectively. Moreover, hydrogen bindings and hydrophobic interactions are the main mechanisms of interaction between KOS and PGM at pH 7.4.

## Conclusions

In summary, two keratins, including KTN and KOS, showed the strong mucoadhesive properties in the present study, and different behaviors in mucoadhesion between KTN and KOS were identified due to their different hydrophilicity, surface charge and terminal groups. The in vivo mucoadhesive capacity in form of gastric retention time of KTN/KOS composite nanoparticles could be controlled by changing the ratios of KTN to KOS, as well as for controlled drug release due to the pH-sensitive feature of KTN. Besides, the drug bioavailability also could be regulated by controlling the gastric retention time and drug release rate of nanoparticles. Furthermore, the mechanisms of mucoadhesion of keratins (KTN and KOS) were firstly investigated in present study. The binding between KTN and PGM is dominated by electrostatic attractions and hydrogen bonding at pH 4.5, and disulfide bond also plays a key role in the interaction at pH 7.4. Unlike KTN, the main mechanism of KOS and PGM interactions is hydrogen bonding at pH 4.5, and hydrogen bonding and hydrophobic interactions s are the main mechanisms of interaction between KOS and PGM at pH 7.4. This study offers an efficient strategy to control the gastric mucoadhesion and drug release of keratin based nano drug delivery systems, and the revelation of mucoadhesive mechanisms of keratins in different pH conditions are helpful for the design and development of mucoadhesive drug delivery systems.

## Additional file

**Additional file 1: Figure S1.** The schematic diagram of the ultrasonic system for the synthesis of KNPs. **Figure S2**. The time-dependent changes in the average size of KNPs within 7 days. **Figure S3**. H&E histological examination of the main organs after oral administration of the KNP-3 for 7 days (original magnification 100 ×).

## Abbreviations

KTN: kerateine
KOS: keratose
PGM: porcine gastric mucin
KNPs: keratin nanoparticles
PK: pharmacokinetic
AFM: atomic force microscopy
ITC: isothermal titration calorimetry
AMO: amoxicillin
SGF: simulated gastric fluid
NEM: *N*-ethylmaleimide
LC: loading capacity
EE: entrapment efficiency
SEM: scanning electron microscope
^131^I: iodine-131
ROI: region of interest
C_max_: maximum plasma concentration
T_max_: maximum plasma concentration
AUC_0–12 h_: area under the plasma drug concentration–time curves from time zero to 12 h
TGA: thioglycolic acid
PDI: polydispersity index
SPECT: single-photon emission computed tomography
