# Supplementary material for: Development of keratin nanoparticles for controlled gastric mucoadhesion and drug release
Source: J Nanobiotechnology. 2018 Mar 19;16:24. doi: 10.1186/s12951-018-0353-2 (PMC5858146; doi:10.1186/s12951-018-0353-2)
Supplement: Supplementary file 1 — Additional file 1: Figure S1. The schematic diagram of the ultrasonic system for the synthesis of KNPs. Figure S2. The time-dependent changes in the average size of KNPs within 7 days. Figure S3. H&E histological examination of the main organs after oral administration of the KNP-3 for 7 days (original magnification 100 ×). [file 12951_2018_353_MOESM1_ESM.docx]

Additional materials

Development of keratin nanoparticles for controlled gastric mucoadhesion and drug release

Zhongjun Cheng ^1,2,3^, Xiaoliang Chen ^4^, Dongliang Zhai ^4^, Feiyan Gao ^1,3^, Tingwang Guo ^1,3^, Wenfeng Li ^1,3^, Shilei Hao ^1,3,^*, Jingou Ji ^2,3,^*, Bochu Wang ^1,3,^*

^1^ Key Laboratory of Biorheological Science and Technology, Ministry of Education, College of Bioengineering, Chongqing University, Chongqing 400030, China.

^2^ College of Chemistry and Chemical Engineering, Chongqing University, Chongqing 400030, China

^3^ Collaborative Innovation Center for Brain Science, Chongqing University, Chongqing 400030, China.

^4^ Department of Nuclear Medicine, Chongqing Cancer Institution, Chongqing 400030, China

*Corresponding authors. Tel.:+86 23 6512 0021; Fax: +86 23 6512 0021.

E-mail address: shilei_hao@cqu.edu.cn (S. Hao); 725_tiger@sina.com (J. Ji) wangbc2000@126.com (B. Wang)

**Additional Materials and Methods**

*Preparation of KNPs:*

KNPs were prepared via the ultrasonic dispersion method based on our previous method, and the schematic diagram of the ultrasonic system for the synthesis of KNPs is shown in Additional file: Fig. S1. Briefly, KTN and KOS were dissolved into ultrapure water with different weight ratios of KTN to KOS, including 100/0 (KNP-1), 75/25 (KNP-2), 50/50 (KNP-3), and 25/75 (KNP-4). 0.15 % keratin solutions (w/v) were injected into the diluted HCl solution (pH 3.0) under sonication via a needle and supplied by a syringe pump with the speed of 0.25 mL/min. The ultrasonication was performed by an ultrasonic cell disruption system (JY92-Ⅱ, Scientz, China), and the power of ultrasonic cell disrupter was selected as 400 W. The resultant nanoparticles were lyophilized by a freeze dryer (230, Modulyod, USA) overnight and stored. In addition, the AMO-loaded KNPs were also prepared as above method, and AMO was added into the keratin solution at a concentration of 0.06 % (w/v).

*Stability test:*

The stability of KNPs was also evaluated by monitoring the particle size within 7 days. The particle size distribution of the KNPs were measured using a Zetasizer (Nano ZS90, Malvern, UK). 0.1 N HCl solution (pH 1.2) was prepared as simulated gastric fluid (SGF), which was used to dilute the samples with appropriate concentrations prior to detection.

*In vivo toxicity test:*

The *in vivo* toxicity of prepared keratins nanoparticles (KNP-3, 380 mg/kg) were also studied by intragastric administration for 7 days. The animals were anaesthetized, and the main tissue organs of the rats were then fixed in 4% paraformaldehyde for histopathologic examination.

**Additional Results**

**Additional materials**

**
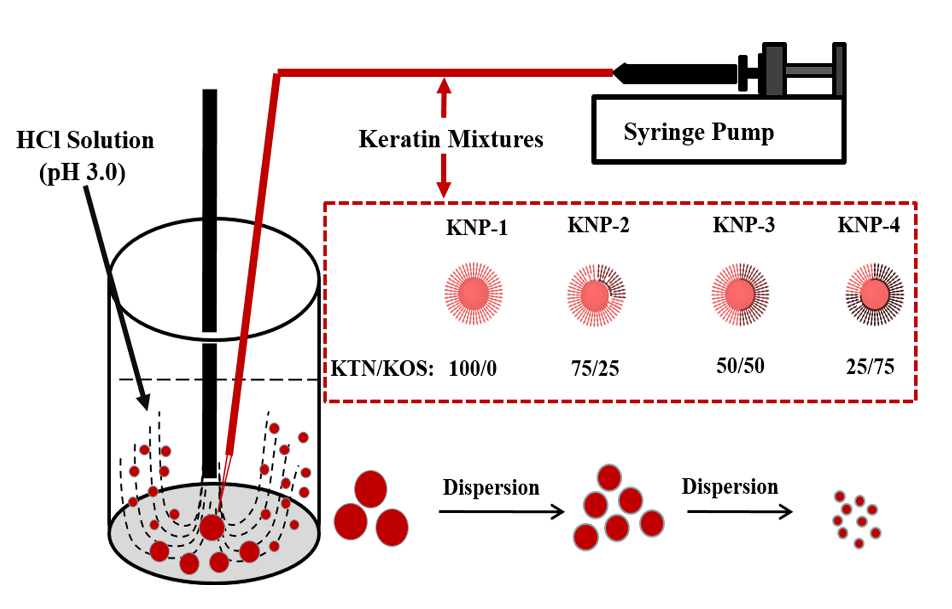
**

Figure S1. The schematic diagram of the ultrasonic system for the synthesis of KNPs

**
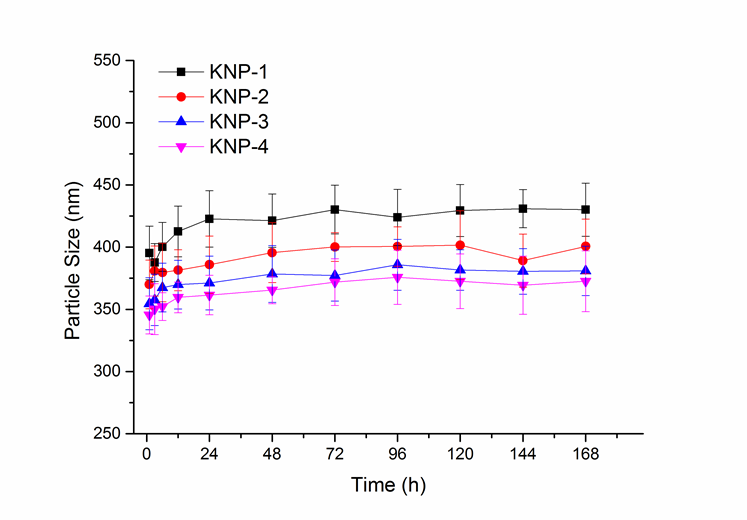
**

Figure S2. The time – dependent changes in the average size of KNPs within 7 days


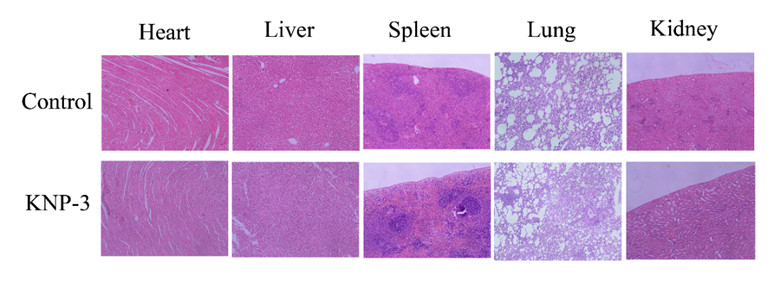


Figure S3. H&E histological examination of the main organs after oral administration of the KNP-3 for 7 days (original magnification 100 ×).
